# Nanox: a miniature mechanical stress rig designed for near-field X-ray diffraction imaging techniques

**DOI:** 10.1107/S1600577516013850

**Published:** 2016-10-18

**Authors:** N. Gueninchault, H. Proudhon, W. Ludwig

**Affiliations:** a MAT – Centre des Materiaux, CNRS UMR 7633, PSL – Research University, BP 87, 91003 Evry, France; b MATEIS, INSA Lyon, CNRS UMR5510, 25 Avenue Jean Capelle, 69621 Villeurbanne Cedex, France; c ESRF – The European Synchrotron, 71 Rue des Martyrs, 38000 Grenoble, France

**Keywords:** miniature stress rig, X-ray diffraction contrast tomography, topotomography, crystal plasticity, *in situ*

## Abstract

Multi-modal characterization of polycrystalline materials by combined use of three-dimensional (3D) X-ray diffraction and imaging techniques may be considered as the 3D equivalent of surface studies in the electron microscope combining diffraction and other imaging modalities. Since acquisition times at synchrotron sources are nowadays compatible with four-dimensional (time lapse) studies, suitable mechanical testing devices are needed which enable switching between these different imaging modalities over the course of a mechanical test. Here a specifically designed tensile device, fulfilling severe space constraints and permitting to switch between X-ray (holo)tomography, diffraction contrast tomography and topotomography, is presented. As a proof of concept the 3D characterization of an Al–Li alloy multicrystal by means of diffraction contrast tomography is presented, followed by repeated topotomography characterization of one selected grain at increasing levels of deformation. Signatures of slip bands and sudden lattice rotations inside the grain have been shown by means of *in situ* topography carried out during the load ramps, and diffraction spot peak broadening has been monitored throughout the experiment.

## Abstract 2

A compact design for a miniature tensile stress rig, compatible with the space and weight constraints imposed by near-field diffraction imaging techniques, is presented. The device can carry tensile loads up to 500 N and is driven by a piezoelectric actuator which can work in a static and dynamic regime up to frequencies of 100 Hz.

## 1. Introduction   

After more than ten years of development, three-dimensional (3D) X-ray diffraction (3DXRD) techniques now routinely provide orientation maps of polycrystalline materials. Far-field variants of 3DXRD give access to grain center of mass and orientation information in sample volumes containing up to thousands of grains. Employing two-dimensional diffraction detectors positioned some hundreds of millimeters behind the sample, they usually provide ample space for sample environment like stress rigs or furnaces and have been used for time-lapse studies of grain rotations (Margulies *et al.*, 2001 ▸), evolution of strain tensors during tensile loading (Martins *et al.*, 2004 ▸; Oddershede *et al.*, 2011 ▸) or for the observation of grain coarsening processes (Wu & Jensen, 2012 ▸). Near-field diffraction imaging techniques, on the other hand, employ high-resolution X-ray imaging detector systems and provide access to spatially resolved orientation maps and 3D grain morphologies. Since the diffracted beams have to be captured on a high-resolution screen positioned a few millimeters behind the sample position, severe space constraints apply to the design of the auxiliary sample environment in this case. For that reason, the majority of studies involving 3D grain mapping coupled with repeated observations of samples as they evolve as a function of strain (King *et al.*, 2008 ▸) or load cycles (Herbig *et al.*, 2011 ▸; King *et al.*, 2011 ▸) were conducted in such a way that the grain microstructure of the sample was mapped without the auxiliary equipment first, whereas subsequent phase contrast observations at increasing levels of strain or fatigue cycles were performed at higher sample-to-detector distances, imposed by the size of the equipment. This procedure is not without problems since it requires 3D image registration of dissimilar volume data sets (phase contrast and diffraction contrast). The minimum distance imposed by the auxiliary equipment may also compromise the optimum settings, even for phase-sensitive imaging techniques (cracks may give rise to strong artifacts in edge-enhanced phase contrast imaging when working at too large propagation distances). A micro-mechanical testing device compatible with the space constraints imposed by diffraction imaging techniques would solve these problems and enable multi-modal observation such as holotomography (Cloetens *et al.*, 1999 ▸) and diffraction contrast tomography (Ludwig *et al.*, 2009*a* ▸) during (interrupted) load tests whilst maintaining the loading conditions throughout the test, since no unmounting/remounting of the sample is required.

In this work we present a compact design for a tensile testing device and show first results obtained during a tensile test on an Al alloy sample. A combination of near-field diffraction imaging techniques was used to study the onset of plastic deformation in a sample made from a binary Al–Li alloy. As crystalline materials are strongly subject to strain localization either under monotonic or dynamic loading (Ewing & Humfrey, 1903 ▸), there is a renewed interest in non-destructive *in situ* analysis of strain fields and lattice rotations in bulk grains, using non-destructive characterization capabilities provided by hard-X-ray diffraction techniques. Indeed, strain localization has been studied by electron microscopy, scanning electron microscopy and electron backscatter diffraction (Abuzaid *et al.*, 2012 ▸), and appears as a precursor and critical physical process in cracking. However, strain localization is intrinsically a 3D phenomenon, depending on the crystal orientation, the grain morphology and the grain neighborhood (Echlins *et al.*, 2015 ▸). With the combination of monochromatic synchrotron X-ray diffraction imaging techniques and tomographic reconstruction methods, these types of studies are now possible for bulk grains, in 3D (King *et al.*, 2010 ▸; Li *et al.*, 2012 ▸; Pokharel *et al.*, 2014 ▸).

3DXRD microscopes are now available at the ESRF, APS, Petra III, CHESS and SPring-8. Resolving local lattice orientations inside a crystal and/or determining average elastic strain tensors of individual grains inside a polycrystalline sample are non-trivial tasks, and several teams are working on different ways to tackle these problems. One can mention the work of Oddershede *et al.* (2011 ▸) at DTU, Denmark, of Suter *et al.* (2006 ▸; see also Li & Suter, 2013 ▸) at Carnegie Mellon and APS, of Miller *et al.* (2012 ▸) at Cornell and CHESS, of Bernier *et al.* (2011 ▸) at LLNL and Viganò *et al.* (2014 ▸) at the ESRF. Taking advantage of well established and optimized experimental setups, one can now consider four-dimensional (time-lapse) studies, capturing the evolution of the microstructure during a mechanical load test. Several near-field diffraction imaging studies have been performed to probe grain coarsening during grain growth or recrystallization (McKenna *et al.*, 2014 ▸), but performing a clean mechanical test without transferring the sample between an external load frame and the experiment is still challenging. For the purpose of tomographic imaging during a load test there are mainly two types of design providing full 360° visibility of the sample, as required for optimum imaging results. In the first case only the sample is rotating during the experiment, *e.g.* the micropress developed for bone studies at European Synchrotron Radiation Facility (ESRF) (Bleuet *et al.*, 2004 ▸) or the RAMS mechanical load frame with air bearings available at CHESS as described by Shade *et al.* (2015 ▸) or, alternatively, designs where the whole frame is rotating like that described by Buffière *et al.* (1999 ▸). The main drawback of these designs is that they are not especially suited to the more stringent space and weight constraints imposed by X-ray diffraction contrast tomography and topotomography. Indeed, during a DCT experiment one has to position the detector at distances comparable with the field of view of the detector, and for a topotomography experiment the entire tomographic sample stage has to be tilted (rocking curve scan) around a second axis, perpendicular to the beam and the tomographic rotation axis. Thus, a specific design is needed to enable repeated observations based on a combination of these near-field diffraction imaging techniques during a mechanical load test. In this paper we propose a design fully compatible with the 3DXRD microscope at the ESRF and fulfilling the above-mentioned requirements for four-dimensional observations including phase contrast tomography, diffraction contrast tomography and topotomography imaging modalities.

These four-dimensional studies are essential to validate micromechanical simulations. But while models are now capable of simulating some of the physical processes, capturing them within the bulk of polycrystalline microstructures is still very challenging. Recent works by Proudhon *et al.* (2016 ▸), Miller *et al.* (2008 ▸) and Oddershede *et al.* (2012 ▸) have shown the possibility of using initial microstructures as determined by 3DXRD techniques as input to predict the evolution of experimental microstructures upon loading. Comparison of these simulations with four-dimensional experimental observations provides unique possibilities for further refinement and optimization of the models used in the simulations.

## 2. Full-field diffraction imaging techniques for polycrystalline materials   

Three-dimensional X-ray diffraction-based tomography techniques combine the classical tomographic approach [acquisition of a set of projection images at different angles, and reconstruction by suitable algorithms like filtered backprojection or algebraic approaches (Kak & Slaney, 1988 ▸)] and Bragg kinematical diffraction. Unlike in absorption micro-tomography (µCT) where contrasts arise from variations of the attenuation coefficient within the material, diffraction-based imaging techniques exploit Bragg diffraction signals from crystalline domains inside the material. Two of these methods are briefly reviewed in the following sections.

### 2.1. Diffraction contrast tomography   

**Figure 1:**
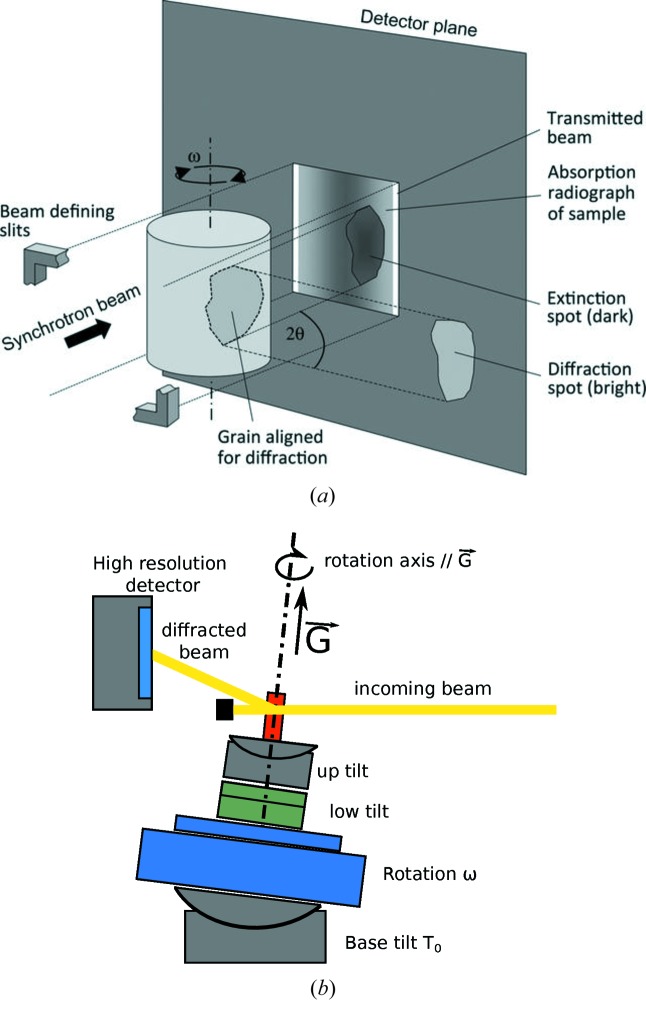
Schematic of a diffraction contrast tomography experiment (from Ludwig *et al.*, 2009*a* ▸) (*a*) and of a topotomography experiment (updated from Ludwig *et al.*, 2007 ▸) (*b*).

Diffraction contrast tomography (DCT) (Ludwig *et al.*, 2009*a* ▸; Reischig *et al.*, 2013 ▸) is a variant of 3D X-ray diffraction (Poulsen, 2004 ▸), using an experimental setup identical to classical absorption micro-tomography. DCT is capable of providing the 3D grain shape, average orientation and elastic strain tensor for every grain of a polycrystal. However, some limitations exist in terms of texture, total number of grains and intragranular orientation spread of the grains which can be analyzed with this technique. Higher values for the combination of these parameters will promote diffraction spot overlap on the detector and will eventually lead to failure of the indexing procedure and not space-filling grain maps. Just like in conventional tomography, the sample is rotated around a single axis and illuminated by an extended, monochromatic beam as shown in Fig. 1 ▸. During the rotation, each grain will fulfill several times the Bragg condition, and the diffracted beams (hereafter called diffraction spots) can be recorded, segmented and indexed using automated analysis procedures developed at the ESRF (Ludwig *et al.*, 2009*b* ▸; Reischig *et al.*, 2013 ▸). DCT has, for instance, been used to track grain growth processes in metals (Johnson *et al.*, 2012 ▸) and ceramics (Syha, 2014 ▸) and has been coupled with phase contrast tomography to observe the process of stress corrosion cracking (King *et al.*, 2008 ▸) and the propagation of fatigue cracks in metals (Herbig *et al.*, 2011 ▸; King *et al.*, 2011 ▸). A recently implemented six-dimensional extension of the reconstruction framework (Viganò *et al.*, 2014 ▸) now enables studies for moderately deformed materials and gives access to the local orientation within the grains (Viganò *et al.*, 2016*a* ▸,*b* ▸).

### 2.2. Topotomography   

In X-ray topography, a two-dimensional projection image of the 3D crystal is recorded on a high-resolution detector system. The technique is sensitive to local variations of the crystal orientation (or deviations from a perfect lattice) and can reveal defects like dislocations, slip bands and stacking faults in rather perfect crystals (Tanner, 1996 ▸). In X-ray topotomography, a crystal is mounted on a dedicated four-circle diffractometer stage (Fig. 1 ▸) and aligned such that the normal of a diffracting lattice plane (a reciprocal lattice vector 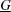) is parallel to the axis of the rotation stage and the grain is located in the center of rotation of the diffractometer stage. To put the grain in the diffraction condition, the tomographic rotation axis 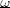 is inclined by the Bragg angle θ by means of the base tilt goniometer 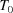. At each rotation position ω a rocking scan covering the width of the crystal reflection curve is recorded by scanning this outer rotation axis [perpendicular to the plane of Fig. 1(*b*) ▸]. By integrating these images of the rocking scan, one obtains a two-dimensional projection topograph of the diffracting grain. The 3D grain volumes of (undeformed) grains can be then reconstructed from a series of tomographic (ω) projection angles, using oblique angle algebraic reconstruction algorithms, available in the *ASTRA* toolbox (Palenstijn *et al.*, 2011 ▸).

This combination of X-ray topography and tomography can be used to extract qualitative information about the 3D arrangement of lattice defects like dislocations in single crystals (Ludwig *et al.*, 2001 ▸) and precipitates in metallic alloys (Ludwig *et al.*, 2007 ▸). Moreover, since the position of the diffraction spot does not change during rotation of a grain aligned for topotomography, one can ‘zoom’ on individual grains using an optimized optical configuration of the detector system or by placing magnifying X-ray optics in the diffracted beam (Simons *et al.*, 2015 ▸).

For structural materials, the combination of DCT and topotomography opens interesting new possibilities for detailed observations of individual grains and grain neighborhoods at the onset of plastic deformation. Defect structures like slip bands and kink bands in metallic alloys can be revealed due to topographic orientation contrast which becomes visible at favorable ω and θ rotation positions during the topotomographic scanning procedure. However, the four-circle diffractometer configuration required for topotomography puts some stringent requirements on the size and weight of the auxiliary sample environment which can be used for this purpose.

## 3. A new compact design compatible with synchrotron X-ray transmission and diffraction imaging   

Generally speaking, integration of an auxiliary sample environment on a synchrotron beamline requires a device to observe severe space constraints, the space around the sample being crowded by detectors, cameras and motorized positioning stages. Geometrical constraints are even more severe for near-field diffraction experiments where the sample needs to be positioned a few millimeters from the high-resolution imaging detector and/or in the center of rotation of the instrument (topotomography). The design presented in the current work targets a tensile specimen, with a cross section typically around 0.5–1 mm^2^.

Specifications were as follows:

(i) Tensile load up to 500 N.

(ii) Apply tensile cyclic load up to a frequency of 100 Hz.

(iii) Load measurement accuracy and stability of 1 N.

(iv) Compatibility with the ID11 diffractometer (*i.e.* maximum 63 mm height from the base plate to the sample position).

(v) The high-resolution imaging detector may approach the rotation center as close as 3 mm.

(vi) Allow 360° rotation for full visibility during tomographic scan acquisition.

A specifically modified piezoelectric actuator (from DSM, USA) was used to fulfill the space constraints imposed by the instrument (63 mm from the mounting interface to the sample/beam position). The actuator is based on a flexure design, has a maximum travel range of 500 µm and can carry up to 650 N of load. A cylindrical quartz tube is used as the load frame which takes place in a mechanical preloading system (fine thread with 0.5 mm pitch). The dog-bone-shaped samples are held in place by two cylindrical pins providing an autoalignement feature with the steel shaft. The shaft was instrumented with a full Wheatstone bridge of semiconductor strain gauges (Texense, France). The strain gauge signal is amplified and conditioned (tunable gain and offset) to generate a 0–10 V signal over 12 bits. This custom load cell can be calibrated on the force range 0–500 N using a classical electromechanical tensile testing machine thanks to machined adaptor parts. Measured load precision and repeatability is 1 N. The actual displacement of the steel shaft is not precisely known and will vary as a function of applied voltage and the effective stiffness of the load chain formed by the metallic housing, quartz capillary, sample, steel shaft and piezoelectric actuator. The determination of the deformation has therefore to rely on digital image (or 3D volume) correlation techniques applied to the X-ray projections (or 3D volumes, respectively). This is further illustrated in §4.2.

The main benefits of using a 1 mm-thick amorphous quartz tube are related to full sample visibility over 360° rotation, the absence of diffraction peaks and its high stiffness and radiation hardness as compared with polymers. The homogeneity and constant absorption allow for high-quality tomographic reconstructions. The weak scattering from the amorphous quartz matrix gives rise to a constant background with smooth spatial variations, which can be easily substracted from the diffraction images.

**Figure 2:**
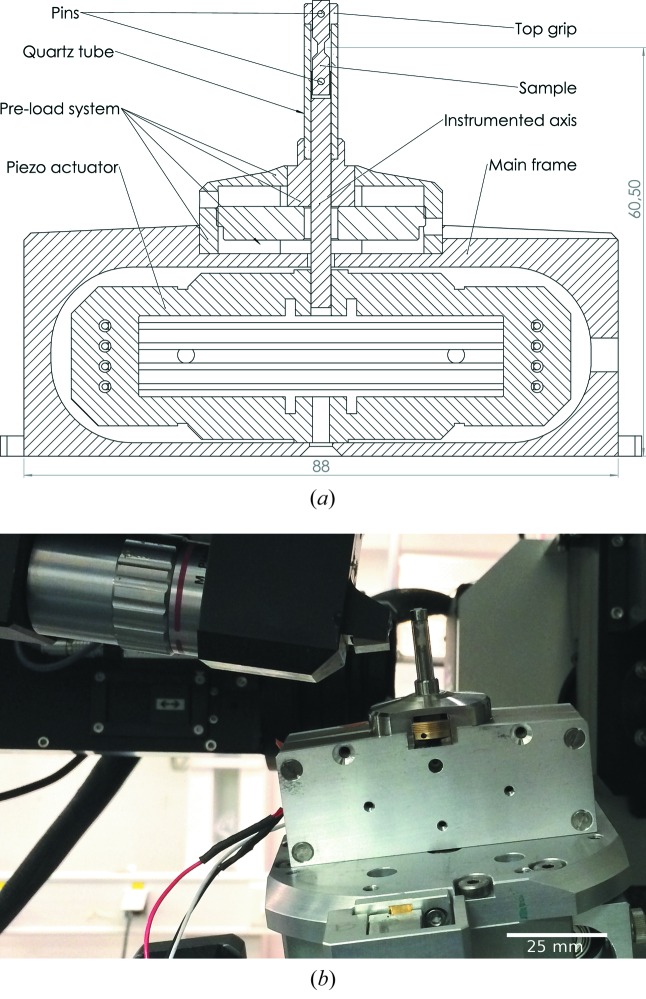
Schematic drawing of Nanox (*a*), and photograph of the device installed on the 3DXRD instrument at ID11, ESRF (*b*).

With the present design (see Fig. 2 ▸), the ID11 high-resolution detector systems can be as close as 2.6 mm to the center of rotation (0.1 mm from the quartz tube), thereby enabling high-spatial-resolution acquisitions with pixel size down to 0.7 µm.

## 4. Application to an *in situ* topotomography experiment on a Al–Li polycrystal   

The Nanox device was tested successfully for a combined DCT and topotomography diffraction imaging experiment during the onset of plastic deformation in a binary Al–Li alloy.

### 4.1. Experimental setup   

The experiment was performed at the ID11 beamline of the ESRF, France. An Al–Li 2.5 wt% multicrystal sample with 0.7 mm × 0.7 mm cross section was mounted in Nanox, itself mounted on the four-circle diffractometer. This instrument is installed in the third experimental hutch, situated at a distance of about 90 m from the in-vacuum undulator insertion device of the beamline. The X-ray beam was monochromated by a bent Laue–Laue Si 111 double-crystal monochromator delivering a relative bandwidth of about 3 × 10^−3^. The energy was set to 41.8 keV.

The sample was carefully mounted into the device, to avoid any initial deformation, and a DCT scan, comprising 3600 equally spaced projections over 360°, was recorded before any loading. The diffraction images were recorded on a 2048 × 2048 pixel high-resolution detector system based on a 50 µm-thick transparent luminiscent screen made from GGG (Martin & Koch, 2006 ▸), optically coupled to an ESRF Frelon camera (Labiche *et al.*, 1996 ▸). The effective pixel size of this system was 1.5 µm.

**Figure 3:**
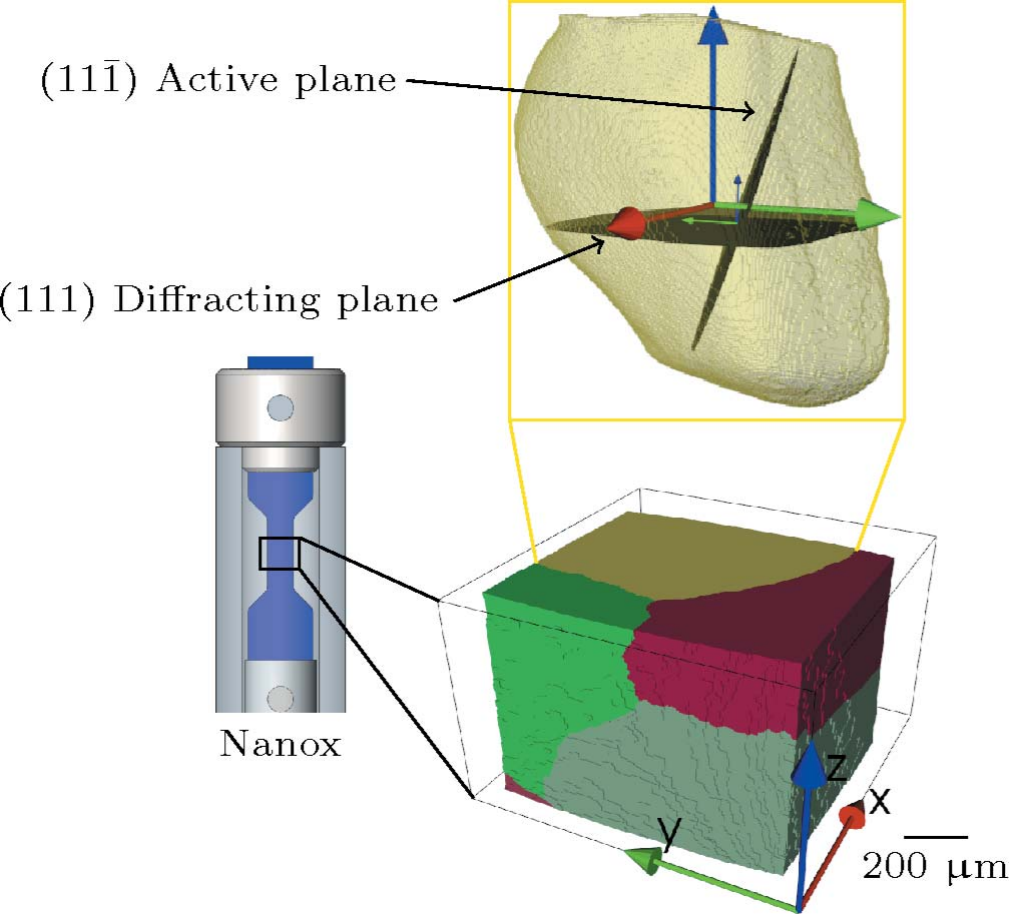
Bottom: 3D rendering of the multicrystal reconstructed by DCT (grain 1 is in yellow). Top: semi-transparent visualization of the outline of grain 1, as obtained from topotomography. The orientation of the diffracting lattice planes and the active slip plane are materialized in black.

Following the usual steps of diffraction spot segmentation, Friedel pair matching and indexing, the orientation and position of all grains in the illuminated sample volume were determined using the DCT analysis code (Ludwig *et al.*, 2009*b* ▸) (Fig. 3 ▸, bottom). Knowing the orientation of the grains, it is then possible to calculate the list of reflections and associated diffractometer tilt angles for aligning these grains and reflections for subsequent characterization by topotomography. Here we selected a grain for which one of the reflections of the {111} family was accessible within the travel range (±20° and ±15° for the upper and the lower tilt motors, respectively) of the ID11 sample goniometer stages.^1^ Once the diffraction vector was exactly aligned with the rotation axis of the tomography stage, the latter was inclined by the corresponding Bragg angle of θ = 3.66° and integrated projection topographs using the previously described scanning procedure (see §2.2) were recorded on a second high-resolution detector system, featuring 1040 × 1376 pixels and an effective pixel size of 0.65 m. The complete experiment was a repetition of the following routine:

(i) The displacement imposed on the sample was incremented by ramping up the voltage of the piezoelectric actuator in constant steps of 1 V (sensitivity: 1 V = 3.33 µm in unloaded condition). This corresponded to a deformation of about 0.22% in the gauge length of the sample. The load ramps were divided into 0.33 µm steps, applied while recording diffraction topographs at ω = 152° and the nominal Bragg angle.

(ii) The width of the reflection curve was updated^2^ and a topotomography scan with 180 projections in ω, covering the full width of the reflection curve in equidistant intervals of 0.1° in θ, was recorded.

**Figure 4:**
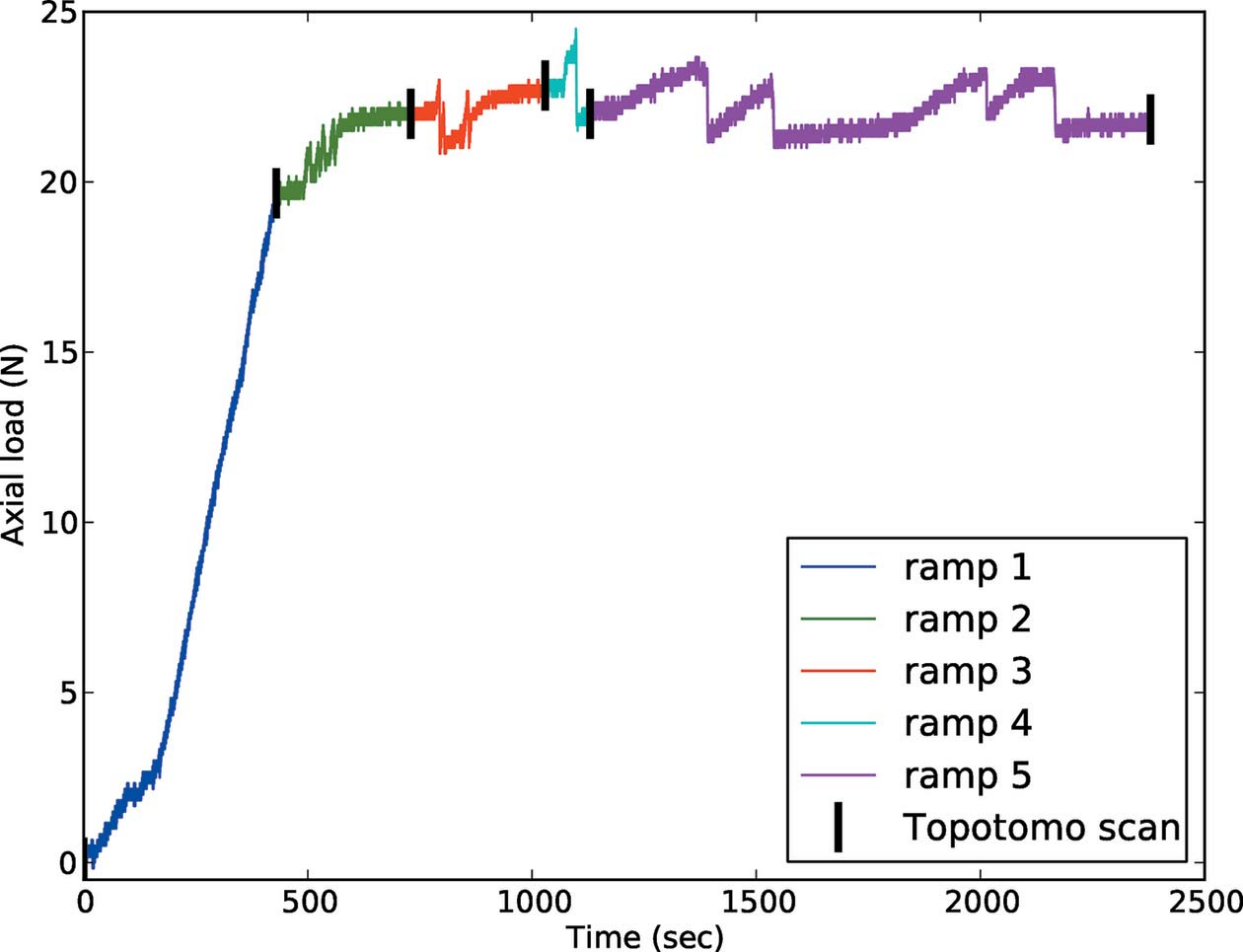
Load history of the sample. Each load ramp is represented by a different color, and the black lines indicate when topotomography scans were performed. For the sake of clarity, the constant load signal during acquisition of the topotomography scans has been removed. Note that ramp 5 itself is composed of four load ramps.

The orientation of the chosen grain resulted in sample goniometer tilt values of 12.95° and 10.68° to align the (111) plane normal collinear to the ω rotation axis of the instrument. The major part of the grain fulfills the diffraction condition at θ = −3.53°. In total, five load ramps were performed covering both elastic and plastic regimes, as shown on Fig. 4 ▸. Observing the loading curve reveals strong instabilities during the load ramps after reaching the elastic limit at about 41 MPa, which is consistent with a critical resolved shear stress around 20 MPa as evaluated from the Taylor factor and the 67 MPa value for this alloy as measured by Rao & Ritchie (1992 ▸). Here the lower yield point is caused by the large grain number 1 which spread over the majority of the cross section, and is favorably oriented for slip (Schmid factor 0.43); see §5.

### 4.2. Inferring strain from image correlation   

**Figure 5:**
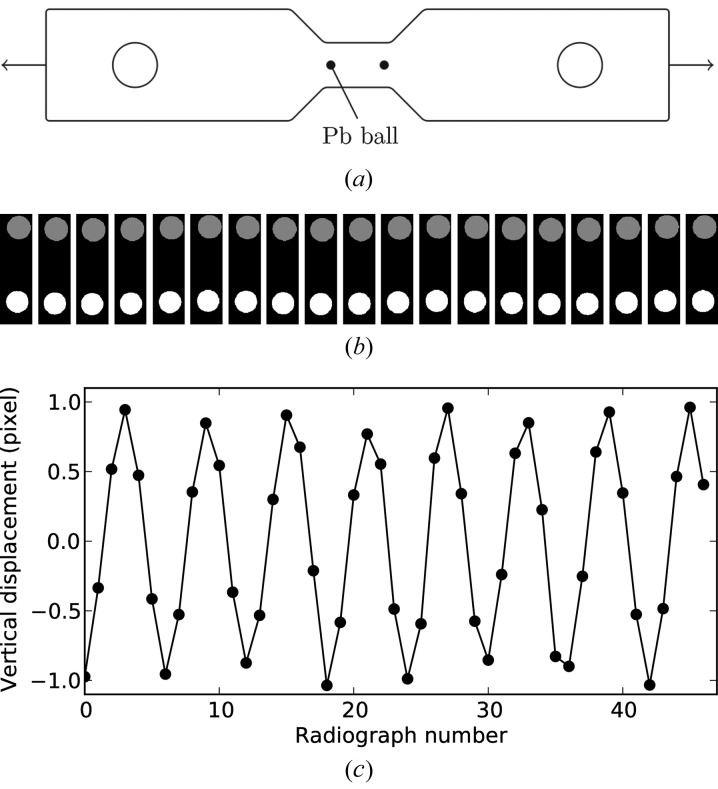
X-ray virtual extensometer. (*a*) Small 316LN tomographic sample with two 50 µm lead balls glued at each end of the gauge length. (*b*) A 100 N cyclic load is applied at 0.1 Hz; X-ray radiographs are recorded every 0.2 s and processed automatically. (*c*) Measured relative displacement in micrometers.

The measurement of macroscopic strain applied to the sample is a critical requirement for the conduction of micromechanical tests. Given the space constraints of the miniature design, we rely on the use of image correlation techniques in order to measure the elongation of the sample in the observation zone. In the simplest case, a virtual extensometer consisting of two absorbing objects [*e.g.* small Pb spheres glued on the sample surface; see Fig. 5(*a*) ▸] can be implemented. The relative position of these objects can be tracked by X-ray radiography as a function of applied load and time. The radiographs are automatically processed by a Python script, determining the center of mass position of the objects and computing the relative displacement (Fig. 5*b* ▸). A test was performed with a 316LN steel sample mounted on the device, and one can use those values to verify the calibration of the system by calculating the apparent Young’s modulus from the measured values for load and deformation. For instance, in the example presented here, the load was 100 N with a section of 0.45 mm × 0.45 mm. The measured displacement was 1.9 pixels over a gauge length of 769 pixels (∊ = 0.0025), which results in a modulus of *E* = 199 GPa (Fig. 5*c* ▸), very close to the known value for this material. These results allowed us to validate this method and generalize it to further experiments. If full tomographic scans are available at each deformation step, digital volume correlation would be a more advanced solution, if the material exhibits sufficient internal contrast (Réthoré *et al.*, 2008 ▸).

## 5. Results and analysis   

In this section, 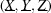 refers to the orthogonal right-handed laboratory basis, 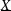 being along the X-ray beam and 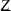 the vertical direction. 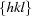, 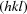 and 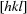 refer to plane families, plane normals and directions expressed in the crystal coordinate system with Millers indices, respectively.

### 5.1. Topographs   

**Figure 6:**
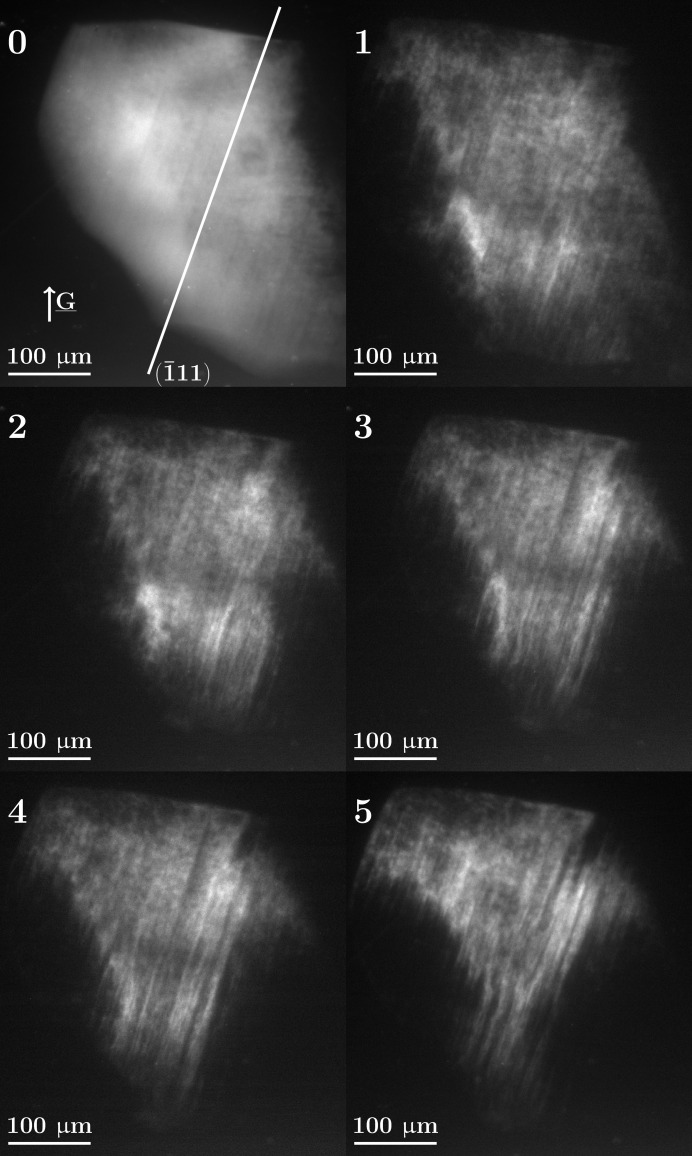
(111) projection Bragg topographs of grain 1 at ω = 152° and θ = 3.80° acquired at each load step. The band-like topographic contrast increases with the applied deformation and increasing subvolumes of the grain rotate out of the Bragg condition. The white line in topograph 0 materializes the intersection of the 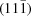 lattice plane with the detector. For this specific ω rotation, this plane is imaged ‘edge-on’ to the detector screen.

As seen in Fig. 6 ▸, in the first scan the grain exhibits only a small intragranular orientation spread (mosaicity) and this orientation spread is observed to increase with increasing levels of plastic deformation. Another interesting observation is the apparition and reinforcement of band-like topographic image contrast, best visible at favorable ω rotation positions, resulting in ‘edge-on’ projections of the active slip plane, as illustrated in Fig. 3 ▸ and in the supporting information. The topographs of Fig. 6 ▸ were recorded in this particular configuration. The inclination of these structures corresponds exactly to the projected trace of the 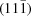 planes in the observed grain and that plane belongs to the slip system with the highest Schmid factor^1^ ^3^, and should therefore be the most easily activated one.

**Figure 7:**
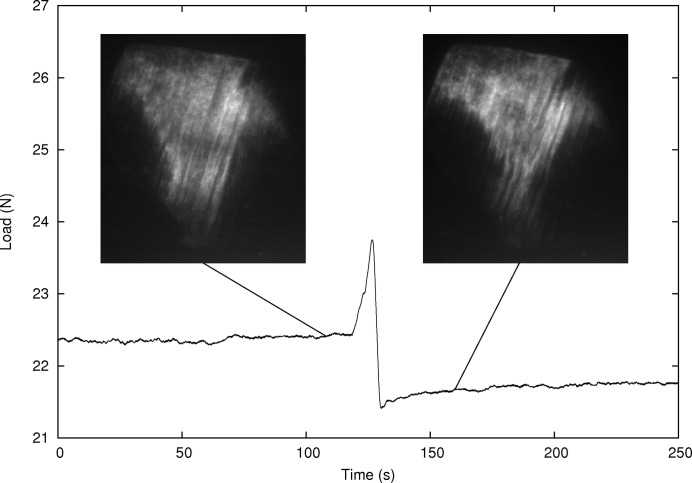
Evolution of the load during the fifth load ramp, and two Bragg topographs at ω = 152° and θ = 3.80°.

Monitoring these topographic contrasts during the actual load ramp reveals another interesting phenomenon: as the load increases, nothing appears to happen, but at one moment the load decreases suddenly by 0.5 N, and at the same time a large part of the grain is no longer in the diffraction condition; see Fig. 7 ▸. This could very well be direct evidence of a ‘Portevin–Le Chatelier’-like effect due to plastic instabilities, and the extinction of the grain is probably due to a spatial rearrangement of dislocation structures, inducing rotation of part of the grain volume.

#### 5.1.1. Reconstructed volume   

At initial stages of deformation, a large fraction of the grain volume occupies a small volume in orientation space, and, for a perfectly aligned setup, this undeformed part of the grain volume would fulfill the Bragg condition at the same base tilt value 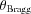 for each of the ω rotation positions. In practice, the plastic deformation and rigid body rotations of the sample upon loading result in a precession of the scattering vector associated with this part of the grain volume around the rotation axis. The highest intensity for a given ω is thus observed at different values of θ which in turn vary as 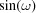. This slight misalignment (∼0.15°) between the rotation axis and the scattering vector has been accounted for in the reconstruction process. As described by Ludwig *et al.* (2001 ▸), topographs were corrected to improve the quality of the reconstruction, by correcting pixel values for constant background. The 3D reconstruction of the main intensity was performed using the *ASTRA* toolbox (Palenstijn *et al.*, 2011 ▸), which can handle arbitrary projection geometries, like the one encountered in topotomography, where the rotation axis is not perpendicular to the directions of the incoming or diffracted beams.

**Figure 8:**
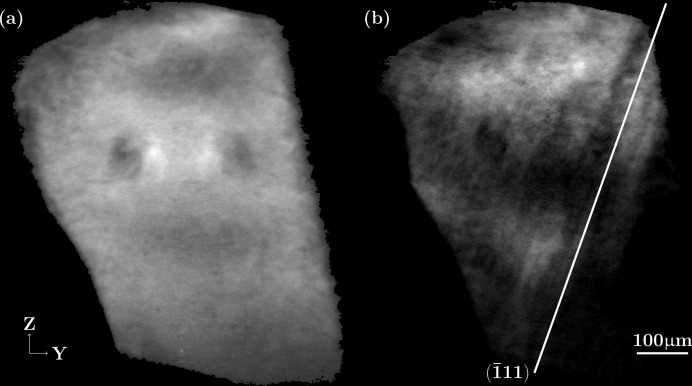
Reconstructed *YZ* slices of the volume, (*a*) before loading, (*b*) after the five load ramps.

The reconstructed grain volumes before deformation and after five load ramps are shown in Fig. 8 ▸. The deformed volume exhibits band-like contrast parallel to the trace of the 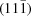 plane, in accordance with the observation in the topographs. Since the reconstruction is based on partially integrated topographs (*i.e.* the image with maximum intensity in the rocking-curve scan), larger parts of the grain, corresponding to subvolumes misoriented by more than 0.1°, are no longer reconstructed.

#### 5.1.2. Rocking curves   

The four-dimensional topotomography experiment consists of taking images of the grain of interest at different ω and θ values for different load (σ) states. The amount of diffracted photons for a given triplet 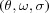 is proportional to the subvolume of the grain fulfilling the Bragg condition, and could be represented as a scalar value *I*, the integrated intensity over the detector range. The width of the *I* = 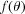 curve for a given 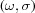 position is a measure of the quality of a crystal (Lübbert *et al.*, 2000 ▸). Contrary to far-field measurements, one cannot directly extract 3D maps or 2D projections of the reciprocal space intensity distribution from images acquired with the near-field acquisition geometry described in this work.^1^ ^4^ On the other hand, the near-field diffraction imaging described in this article allows for direct identification of the active slip system from the topographic (orientation) contrast, visible in a sub-set of the projection images. Note that the visibility of this contrast varies as a function of the rotation angle^2^ ^5^ and reaches a maximum for ω rotation positions close to the ‘edge-on’ configuration depicted in Fig. 3 ▸.

**Figure 9:**
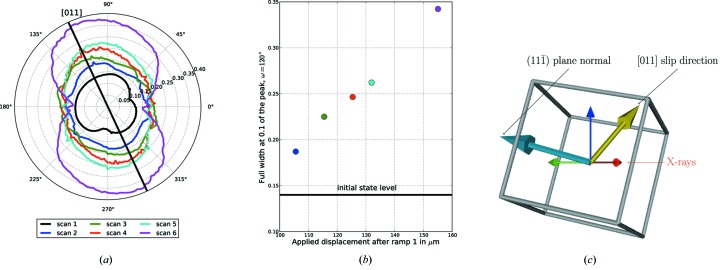

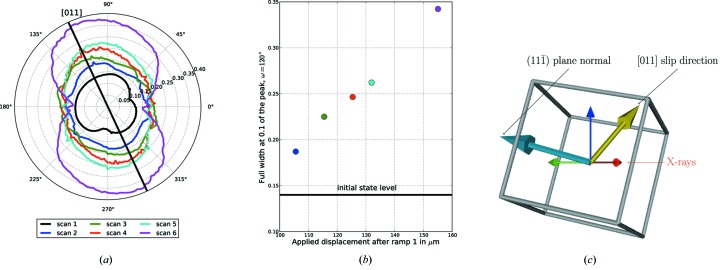
(*a*) Full width at 10% maximum as a function of ω and of the scan. The black line indicates when the 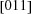 direction of the crystal is within the *XZ* plane. (*b*) Width evolution at ω = 120° as a function of the scan. (*c*) Crystal configuration at ω = 120°. 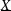 is along the beam while 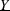 is along the base tilt axis 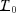. The beam direction, the vertical direction and the slip direction (yellow arrow) are coplanar.

We propose a simplified analysis, whereby plotting the full width at 10% of the maximum of the reflection curves as a function of ω we determine the convex hull of the reciprocal space intensity distribution (integrated along the strain direction). The resulting contours have an elliptical shape and are plotted for each ω and each load state in Fig. 9 ▸. The ellipsis tends to widen in a specific direction (at a preferential ω value). This ω value corresponds to the configuration where the slip direction 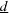 lies in the plane 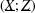, being the direction of the incoming X-rays beam, and 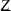 being the vertical axis. In other words, at this particular ω, the slip direction 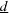 of the active slip system is included in the 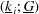 plane, with 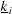 the incoming beam direction and 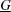 the reciprocal lattice vector. This widening of the ellipsis shows a continuous increase when plotted in this particular direction (Fig. 9 ▸). In this plot, the initial 100 m displacement corresponds to the clearance of the mechanical play between the specimen and the loading pins.

The width of the rocking curves is directly linked to the mosaicity in the crystal. As the load increases, dislocation densities increase too, both at grain boundaries to accommodate the strain and in the grain bulk (dislocation forest and dislocations pile-ups). Stress fields around these dislocations structures generate crystal rotations, which modify locally diffraction conditions as explained by Hull & Bacon (1984 ▸). Depending on the type (screw or edge) and the Burger’s vector of the dislocation, the impact on fulfilling Bragg’s law will not be equivalent. An edge dislocation will bend the crystal, and generate a lattice rotation field, around its line vector. The widening of the rocking curve along the [011] direction observed in Fig. 9 ▸ could be seen as an indirect observation of a pile-up of edge dislocations with the Burger’s vector perpendicular to this [011] slip direction.

## 6. Discussion   

As diffraction imaging techniques give access to the 3D grain microstructure at the micrometer length scale, it is possible to create realistic meshes of polycrystalline microstructures as encountered in common structural materials like metals and their alloys. Combining real microstructure (image) based numerical computations and four-dimensional observations of the same microstructure evolving as a function of strain or temperature offers unique possibilities to corroborate predictions from material models like crystal plasticity or phase field. Note that conventional 3D observations based on destructive serial sectioning techniques can only provide a snapshot of the state of the sample at a given time. Regarding plastic deformation of polycrystalline aggregates, it is well known that misorientations between grains lead to strain incompatibilities and result in a complex heterogeneous response of the material to mechanical loading (Ashby, 1970 ▸). Access to local crystallographic orientation would allow advanced models of material constitutive behavior to be tested and validated, like strain-gradient plasticity (Forest & Guéninchault, 2013 ▸), where dislocations densities are linked to the local lattice rotations (Nye, 1953 ▸). Moreover, if the microstructure provides sufficient contrast in the reconstructed tomographic image, the crystallographic information can be complemented with 3D deformation fields, *via* digital volume correlation. Using phase-sensitive imaging techniques, one can also follow the evolution of damage (porosity, cracks) and again these observations can be compared with model predictions. Last, mechanical computations could be used to constrain the solution space and to improve the quality of the tomographic reconstruction in diffraction experiments.

More efforts are needed to increase experimental capabilities to directly compare with simulations of crystal deformations. Indeed, the latter can routinely compute both local and global variables (*e.g.* average stress tensor of a grain and local stresses inside the grain), whereas it is currently possible to access the local (intragranular) strain and stress variations with the help of full-field diffraction imaging techniques.^3^ ^6^

## 7. Conclusion   

We have presented a compact design for a miniature tensile stress rig, compatible with the space and weight constraints imposed by near-field diffraction imaging techniques. The device can carry tensile loads up to 500 N and is driven by a piezoelectric actuator which can work in a static and dynamic regime up to frequencies of 100 Hz.

The design allows for minimum sample-to-detector distances of 2.6 mm and to position the sample in the center of rotation of the four-circle 3DXRD instrument at beamline ID11. Whereas the former is a prerequisite for full-field grain mapping in small grained materials (10–30 µm), the latter allows for observations of individual grains and grain neighborhoods by means of topotomography, revealing the early stages of plastic activity and associated diffraction peak broadening. The capacity to combine diffraction contrast tomography (3D mapping of grain microstructure), topo­tomography (high-resolution mapping of individual grains, localized plastic activity, peak broadening) and holotomography (internal surfaces, crack initiation) on the same instrument and without unmounting the sample opens new perspectives for studying the early stages of plasticity and damage in polycrystalline materials (*e.g.* formation of persistent slip bands).

As an illustration of the combined multi-modal observation capabilities of the device, we present *in situ* observations of plastic instabilities, slip band formation and diffraction peak broadening at increasing levels of tensile strain in a selected grain of a grain-mapped multicrystal prepared from Al 2.5 wt%–Li alloy.

## Supplementary Material

Full set of radiographs over 360 degrees integrated over the whole theta range (about 1 degree) after load ramp 5. DOI: 10.1107/S1600577516013850/ie5166sup1.avi

Full set of radiographs over 360 degrees integrated over +/- 0.05 degrees of the peak of the rocking curve. DOI: 10.1107/S1600577516013850/ie5166sup2.avi

## Footnotes

1 Given these tilt ranges, most grains can only be aligned for a limited sub-set of *hkl* reflections.

2 With increasing deformation, the orientation spread in the grain increases and the angular range of the rocking scan has to be enlarged in order to cover the full grain volume.

3 The slip system  has a Schmid factor of 0.43, the highest for the {111} family of this grain.

4 One could envisage the reconstruction of 2D projections from a series of integrated near-field intensity profiles  acquired at different ω rotation positions. This leads to an inverse problem, similar to the one of image reconstruction from projections, and has not been further considered in this work.

5 The lattice rotation giving rise to this orientation contrast may very well be linked to the presence and accumulation of dislocations on different slip systems, interacting and piling up in the vicinity of the band structures created by the dominant system.

6 Polychromatic and monochromatic scanning microdiffraction techniques can provide access to this type of information but currently involve acquisition times which are incompatible with *in situ* studies of extended 3D sample volumes.
